# Supplementary material for: Detection of Hybrids in Willows (Salix, Salicaceae) Using Genome-Wide DArTseq Markers
Source: Plants (Basel). 2024 Feb 26;13(5):639. doi: 10.3390/plants13050639 (PMC10935248; doi:10.3390/plants13050639)

Figure S2. Examples of the *Salix alba* samples. Parts of herbarium specimens (deposited in OL). All samples of *S. alba* in this study are morphologically similar, species exhibit limited morphologic variation in leaf-shape. All collected by R. J. Vašut. Arrows indicate samples in the STRUCTURE bar plot (see Figure 8).

(a) ID #034 – natural (Czechia).

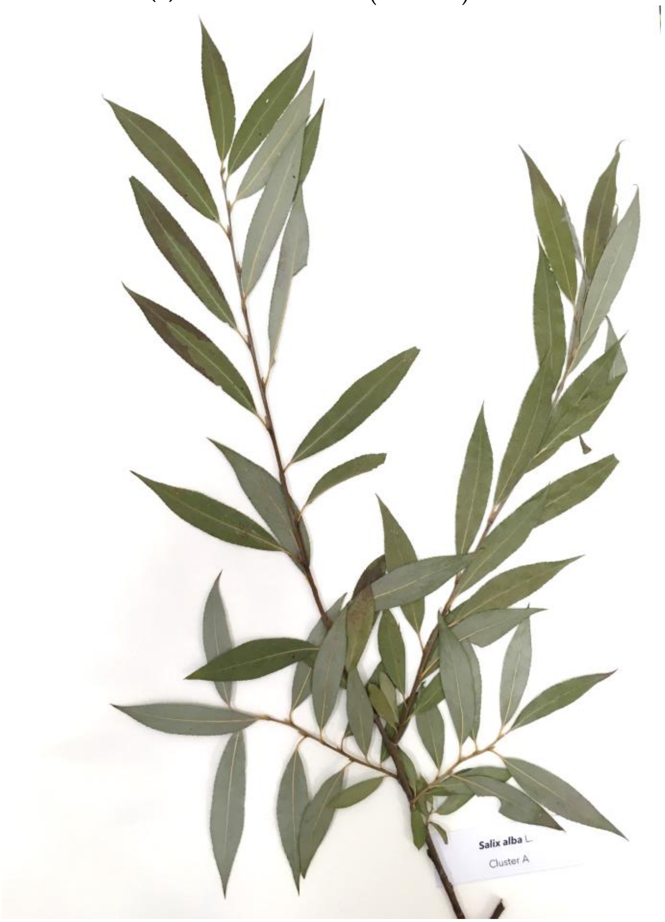

(b) ID #A025 – natural (Czechia).

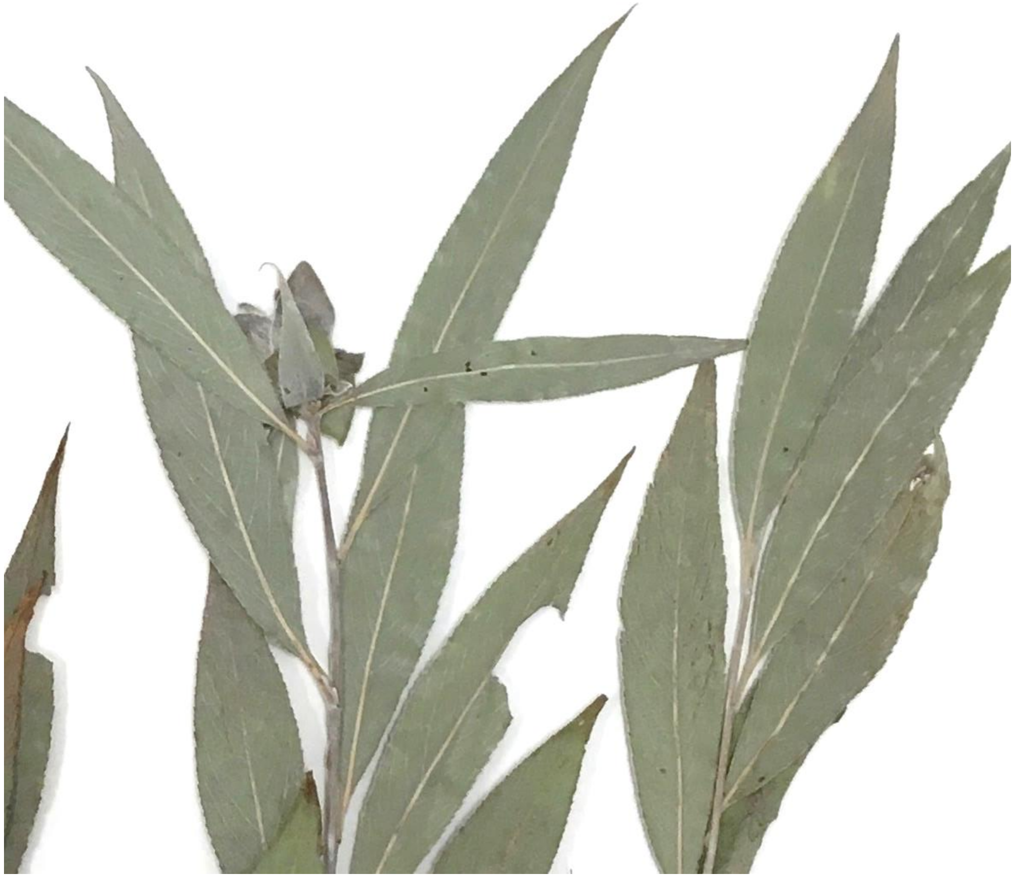

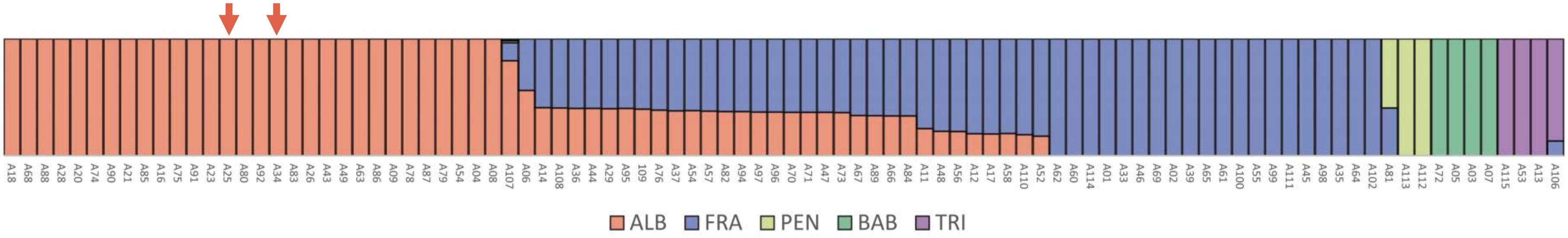

Figure S3. Examples of the *Salix fragilis* samples. Parts of herbarium specimens (deposited in OL). Samples of *S. fragilis* varies in leaf-shape, shown are narrow-leaved morphotypes (resembling the S. fragilissima Host). Branches of this species are always glabrous, never hairy (not visible form images). All colected by R. J. Vašut. Arrows indicate samples in the STRUCTURE bar plot (see Figure 8).

(a) ID #035 – natural (Czechia).

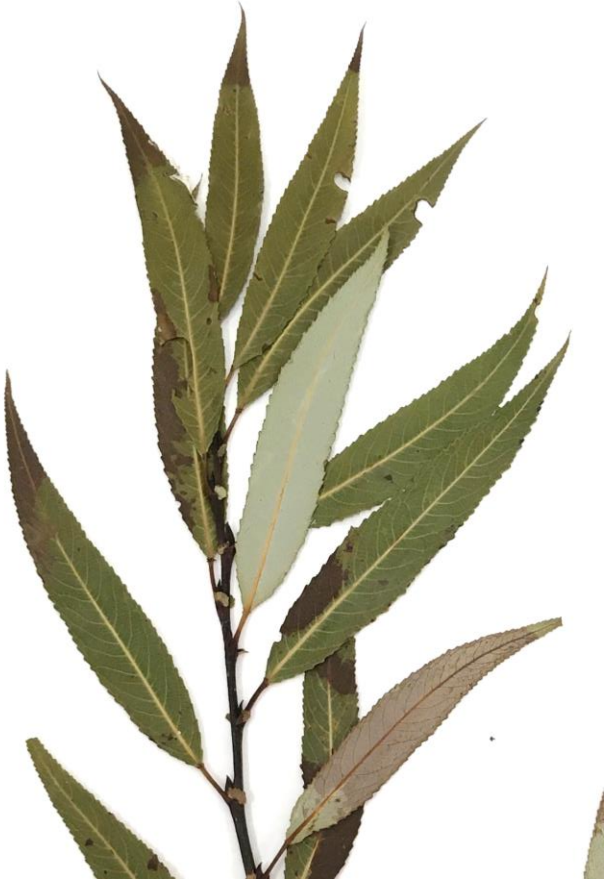

(b) ID #035 – detail of leaf.

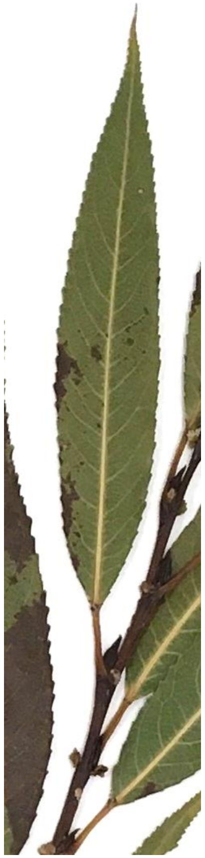

(c) ID #A033 – natural (Czechia).

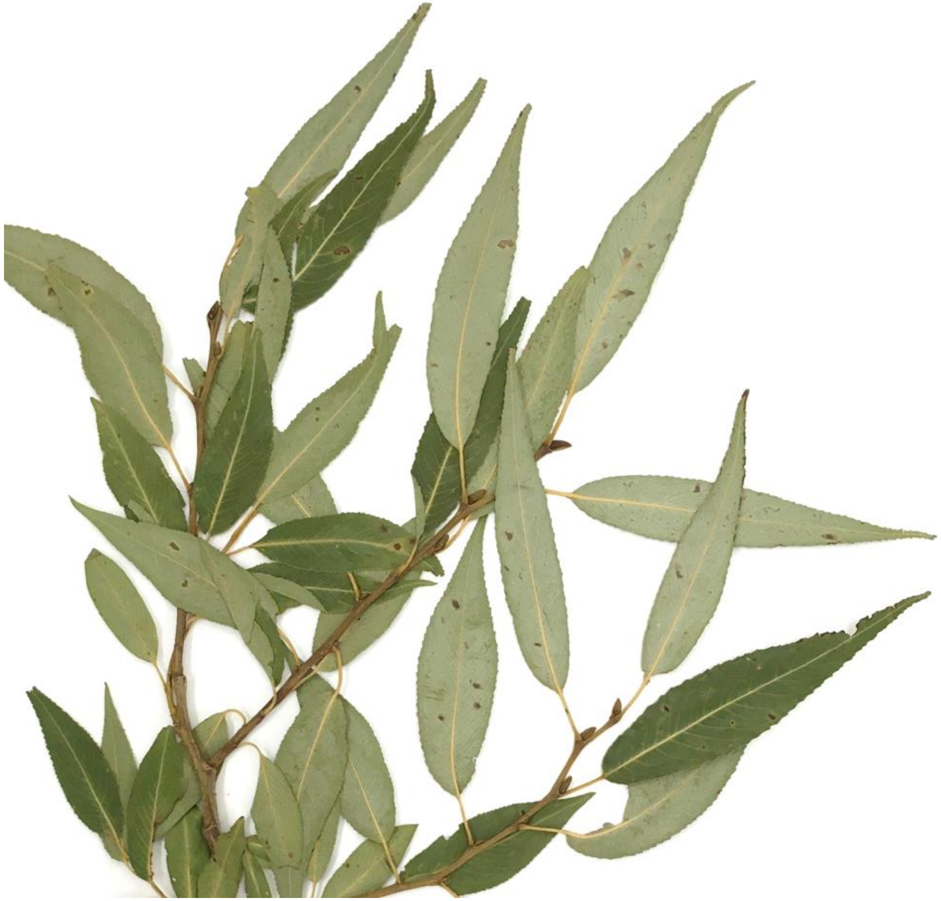

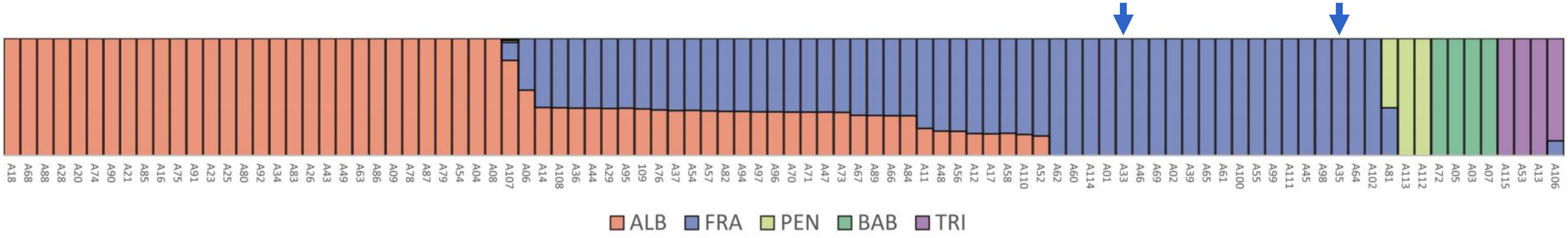

Figure S4. Examples of morphotypes of the *Salix* ×*rubens* (F_1 hybrids of *S. alba* × *S. fragilis*). Scan of **(a)** branch from living plant from the cultivated willow collection, scanned by J. Weger (JW); or **(b)** parts of herbarium specimens (deposited in OL, collected by R. J. Vašut - RJV). Arrows indicate samples in the STRUCTURE bar plot (see Figure 8).

(a) ID #096 – natural (Czechia), JW (fraBrt450)

(b) ID #109 – natural (Slovakia), RJV.

(c) ID #A108 – natural (Poland), RJV.

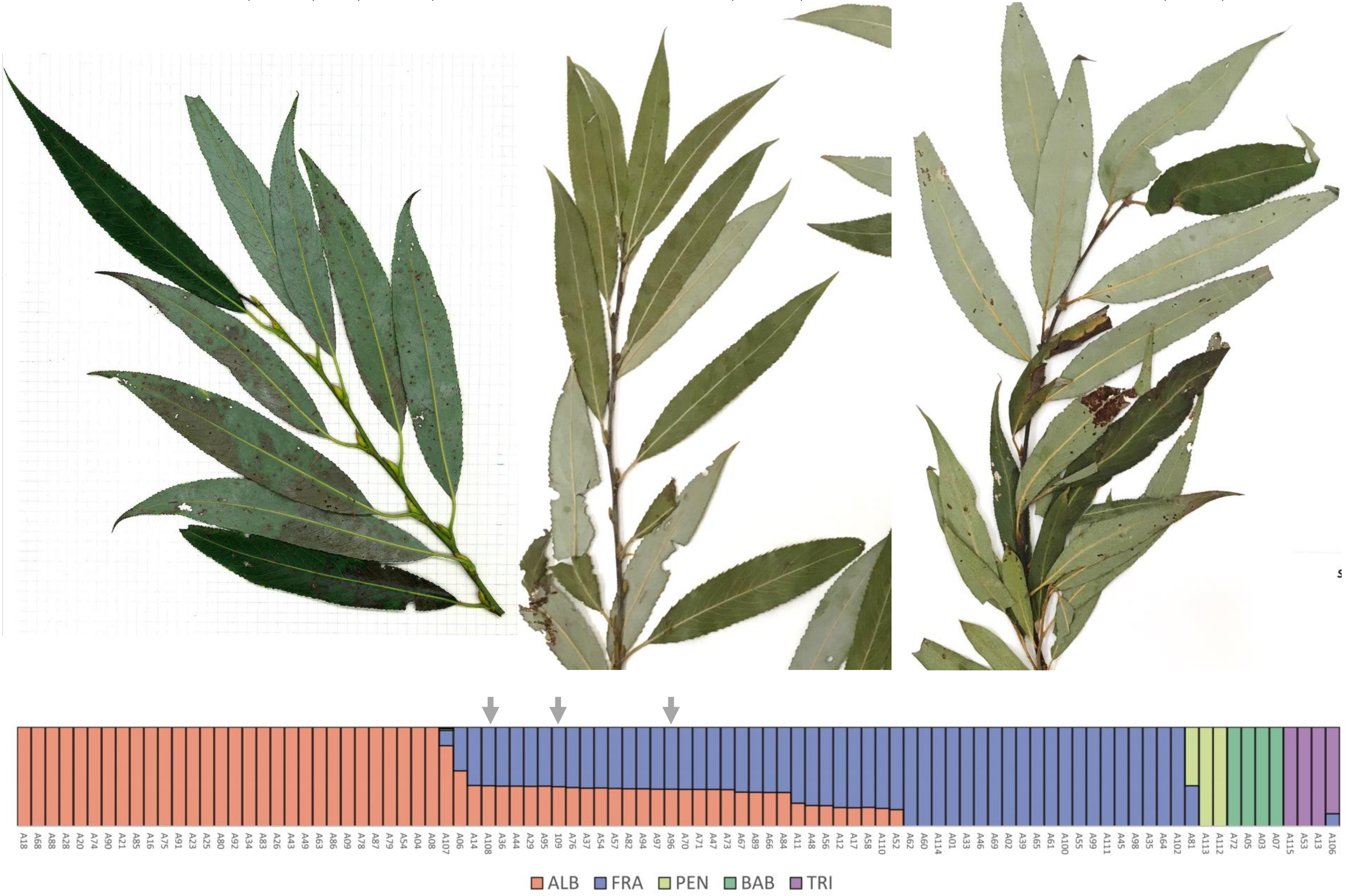

Figure S5. Examples of morphotypes of the *Salix* ×*rubens* × *S. fragilis* backcross hybrids. Morphotypes are morphologically variable, often hard-to-recognize form the *S. fragilis*. Samples have always at least slightly hairy twigs (not visible from images, in binocular only). Arrows indicate samples in the STRUCTURE bar plot (see Figure 8).

(a) ID #A058 (natural).

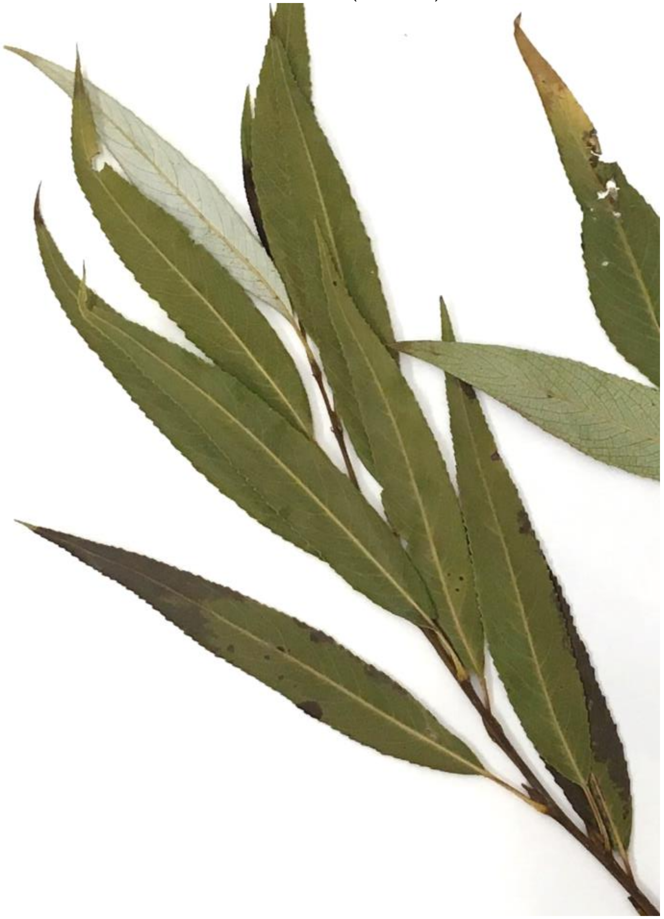

(b) ID #A048 (natural)

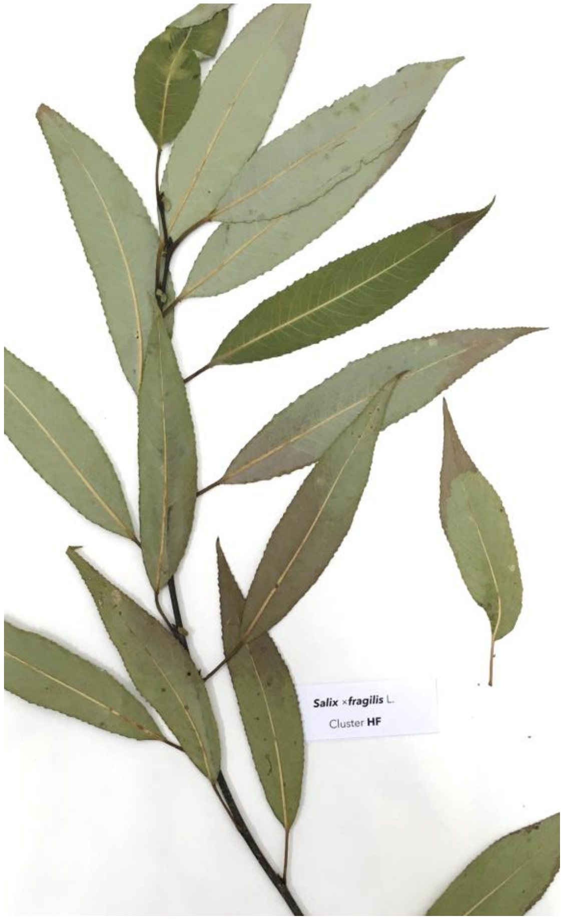

(c) ID #A052 (natural)

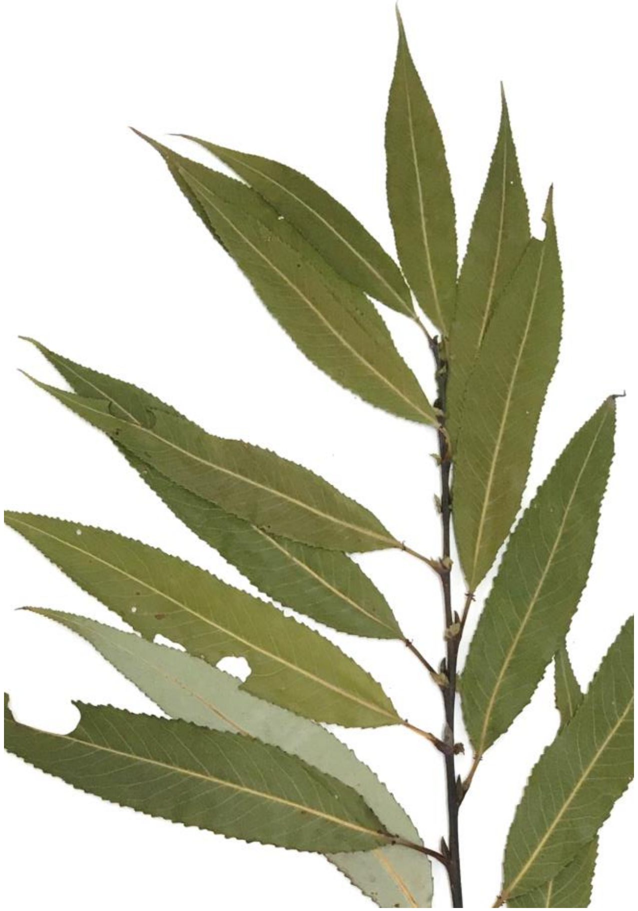

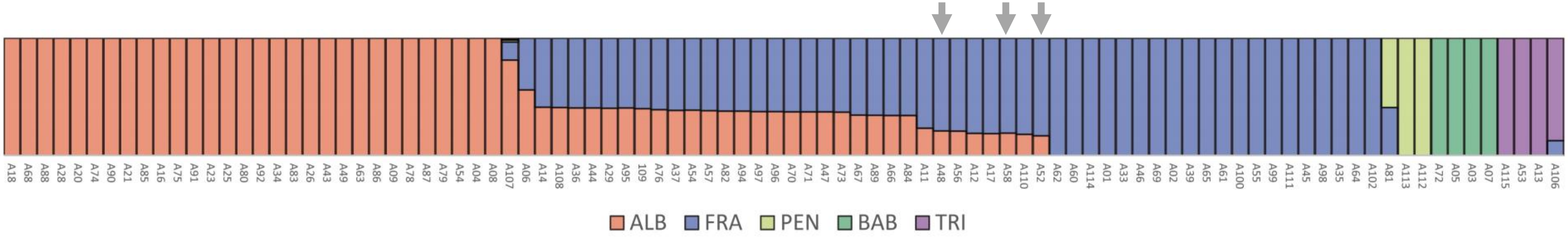

Figure S6. Examples of the *Salix pentandra* sample. Sample ID #A112, collected in the field by R. J. Vašut, part of herbarium specimen deposited in OL. Arrow indicates the sample in the STRUCTURE bar plot (see Figure 8).

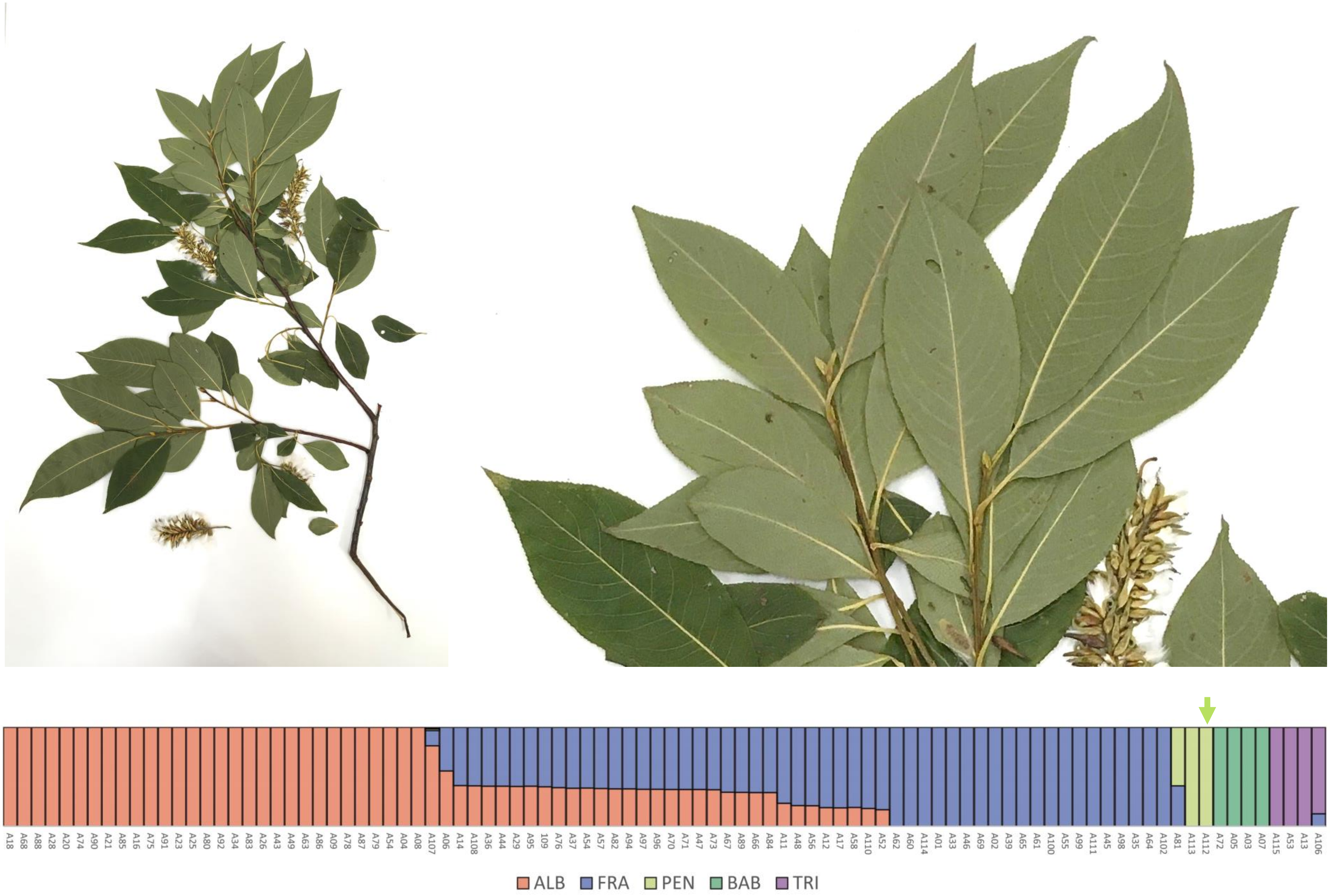

Figure S7. Part of the herbarium specimen of the *Salix babylonica* 'Babylon' sample. Sample ID #A005, cultivated in the campus of Faculty of Sciences of the Palacký University. Collected by R. J. Vašut, deposited in OL. Arrow indicates the sample in the STRUCTURE bar plot (see Figure 8).

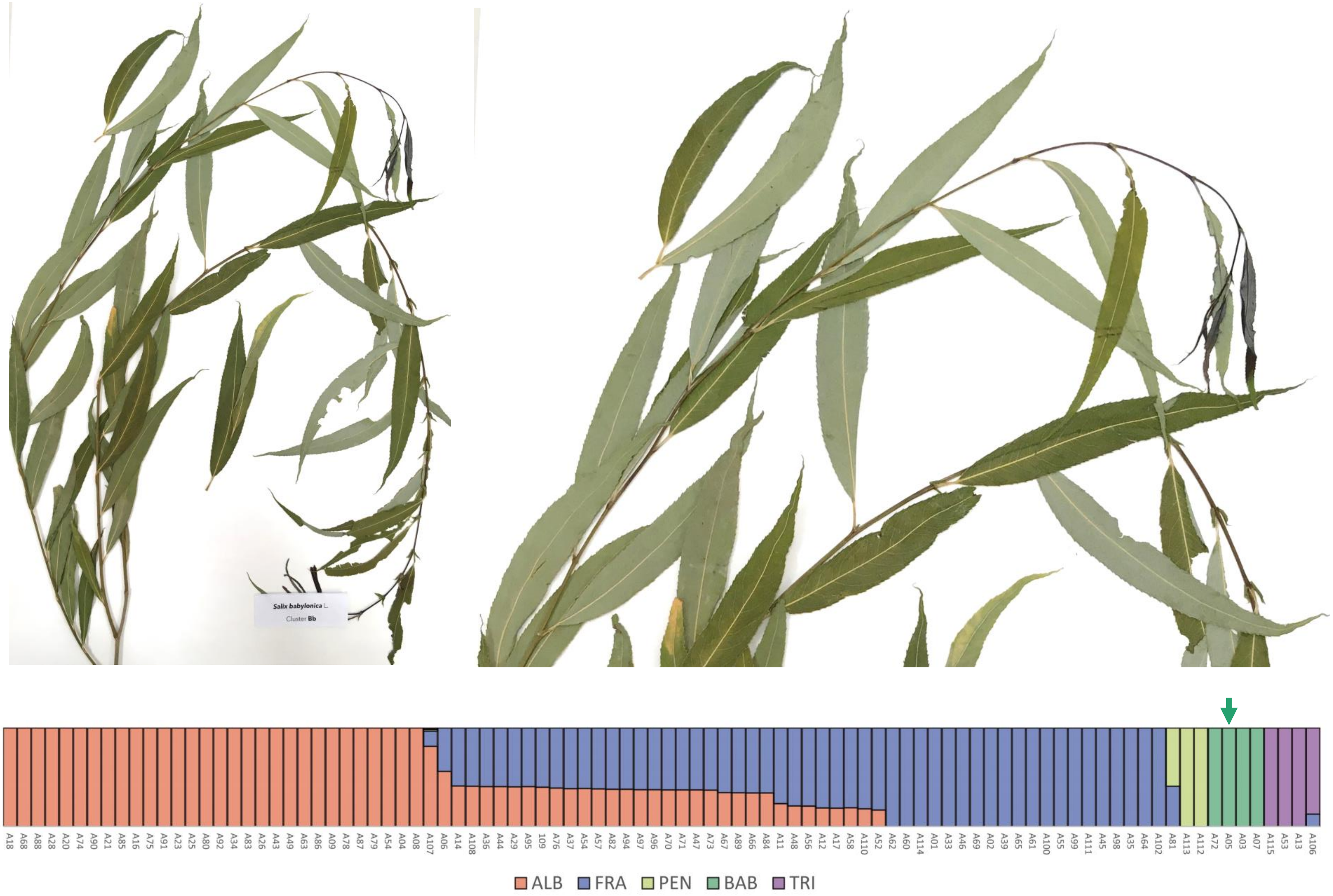

Figure S8. Part of the herbarium specimen of the *Salix triandra* f. *triandra* sample. Sample ID #A053, collected in in the field by R. J. Vašut, deposited in OL. Arrow indicates the sample in the STRUCTURE bar plot (see Figure 8).

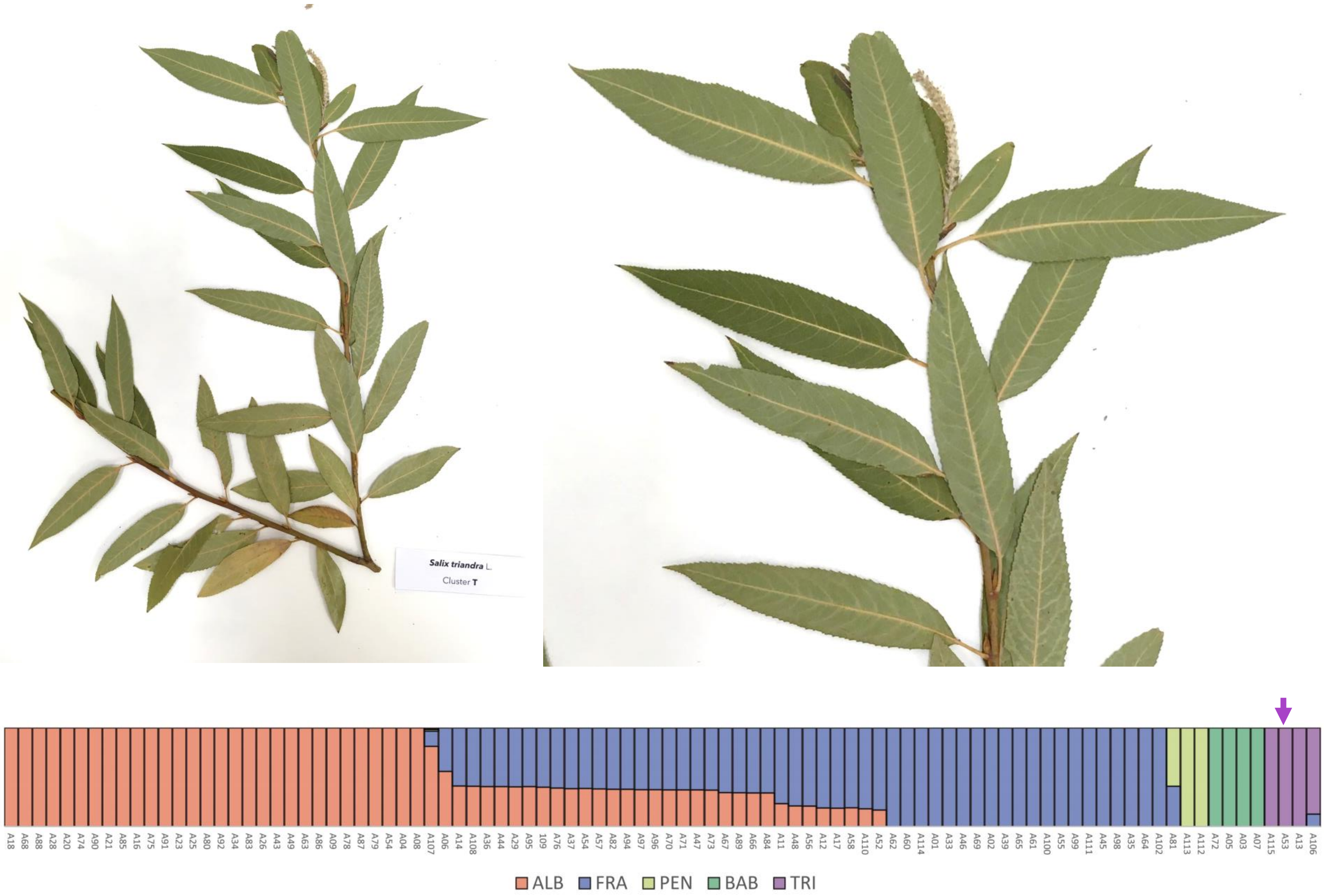

Figure S9. Herbarium specimen of the *Salix fragilis* × *S. pentandra* F_1 hybrid. Sample ID #A081, scan from plant cultivated in willow collection by J. Weger (rubVes-391). Arrow indicates the sample in the STRUCTURE bar plot (see Figure 8).

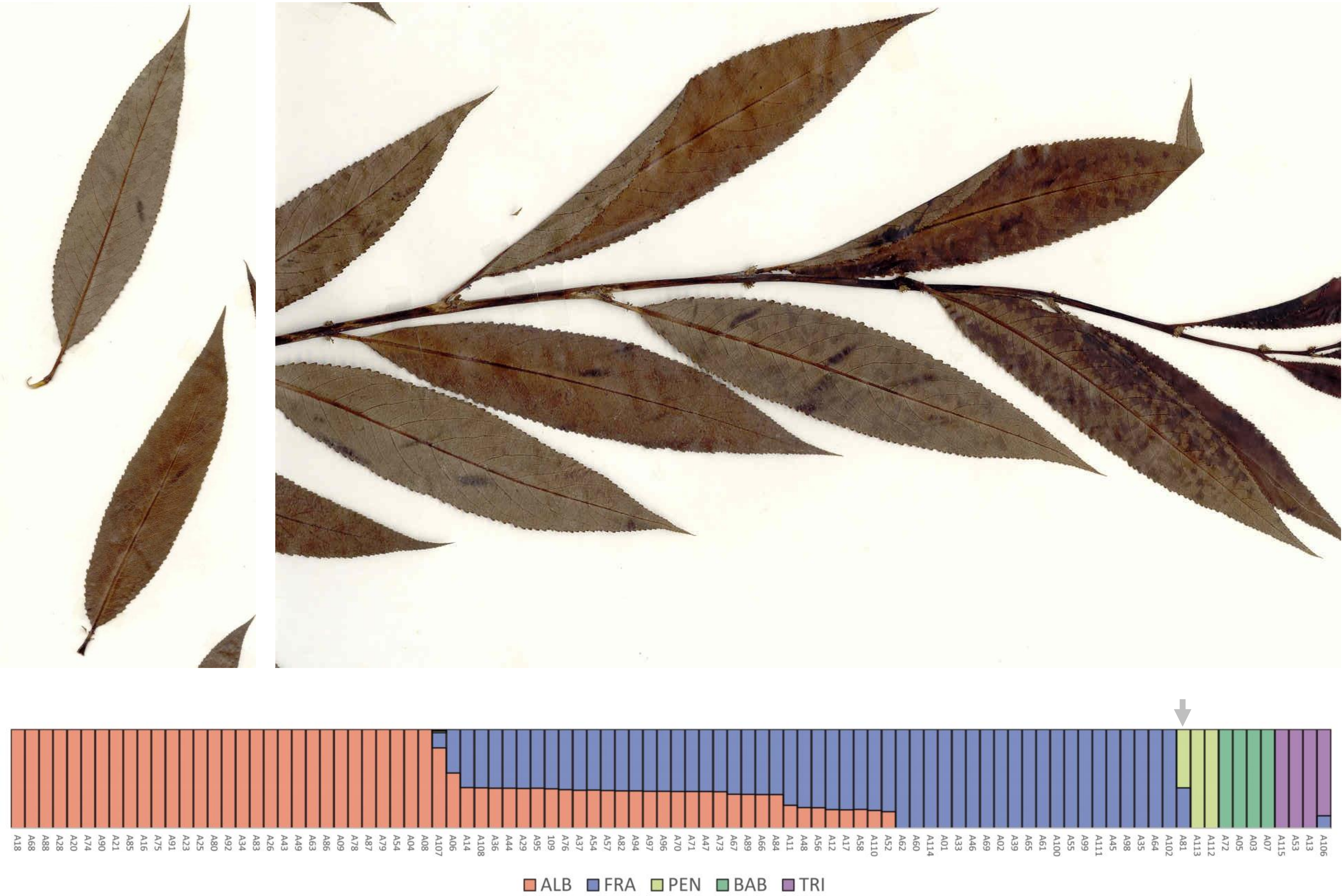

Figure S10. Examples of herbarium specimens of samples of the *Salix* subgen. *Vetrix* hybrids—hybrids of *S. caprea* × *S. viminalis* (*S.* ×*smithiana*). (a) typical morphotype of *Salix* ×*smithiana*, sample ID #G74; part of twig and detail of leaves; (b) another morphotype of the hybrid, sample ID #G70, part of twig and detail of leaves. Herbarium specimens deposited in OL. Arrows indicate samples in the STRUCTURE bar plot (see Figure 8).

(a) ID #074 – (cultivated, JW).

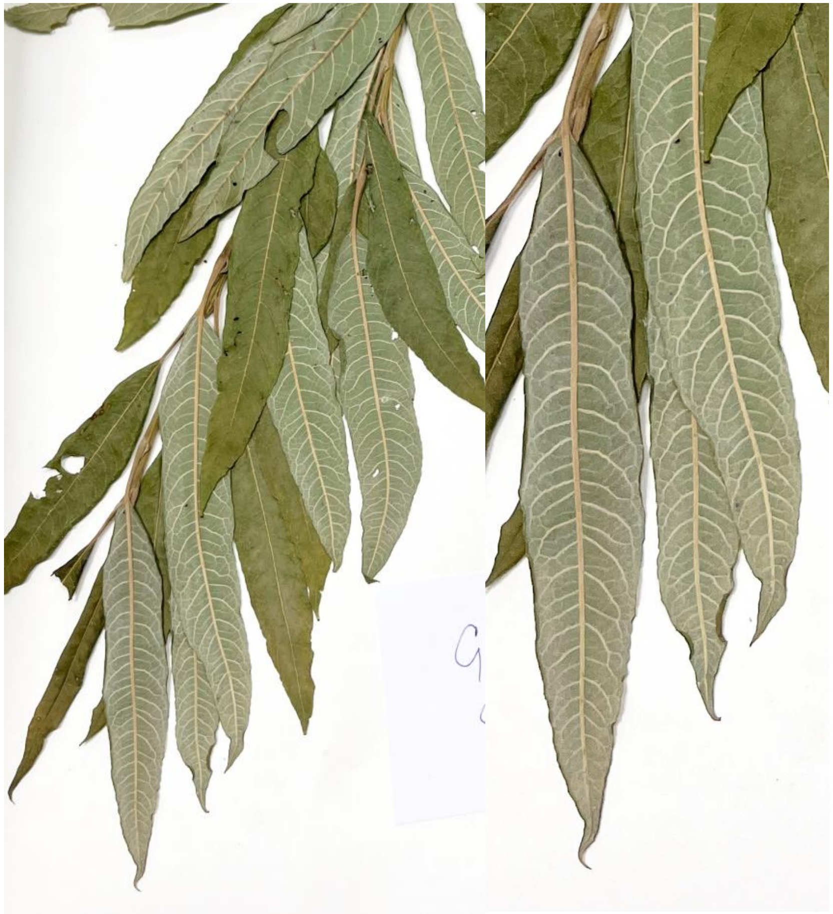

(b) ID #G70 – (cultivated, JW).

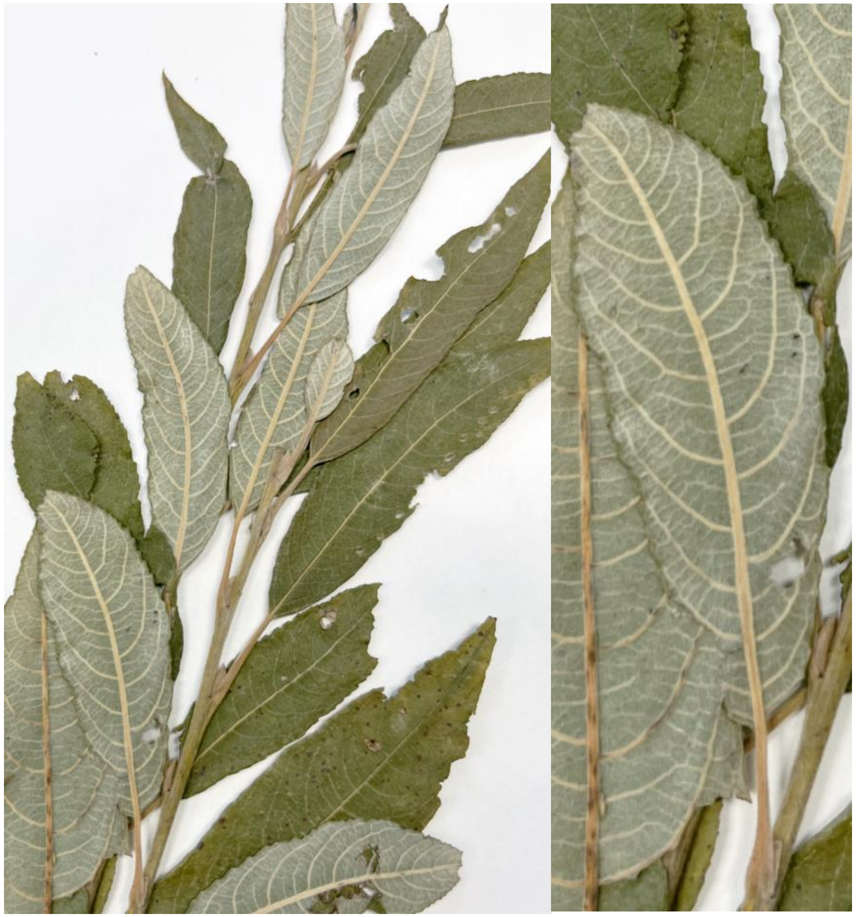

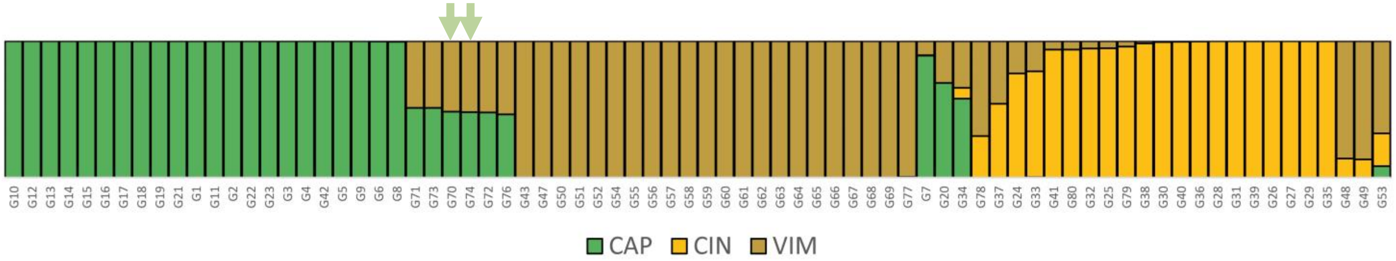

Figure S11. Examples of herbarium specimens of samples of the *Salix* subgen. *Vetrix* hybrids—*Salix viminalis* and the hybrid of *S. cinerea* × *S. viminalis*. (a) distinct morphotype of *S. viminalis*, sample ID #G77 (cultivated as *S.* „*rossica*“ × *S. viminalis*); (b) hybrid *S. cinerea* × *S. viminalis*, sample ID #G78 (cultivated as *S. dasyclados* x *S. viminalis*). Herbarium specimens deposited in OL. Arrows indicate samples in the STRUCTURE bar plot (see Figure 8).

(a) ID #077 – (cultivated, JW).

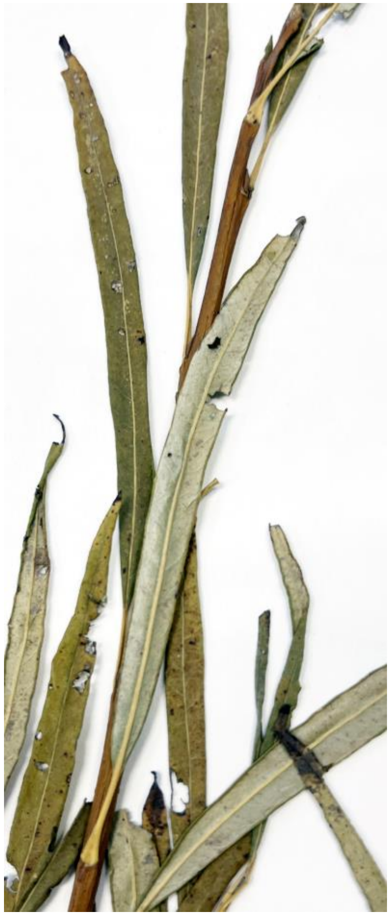

(b) ID #G78 (cultivated, JW).

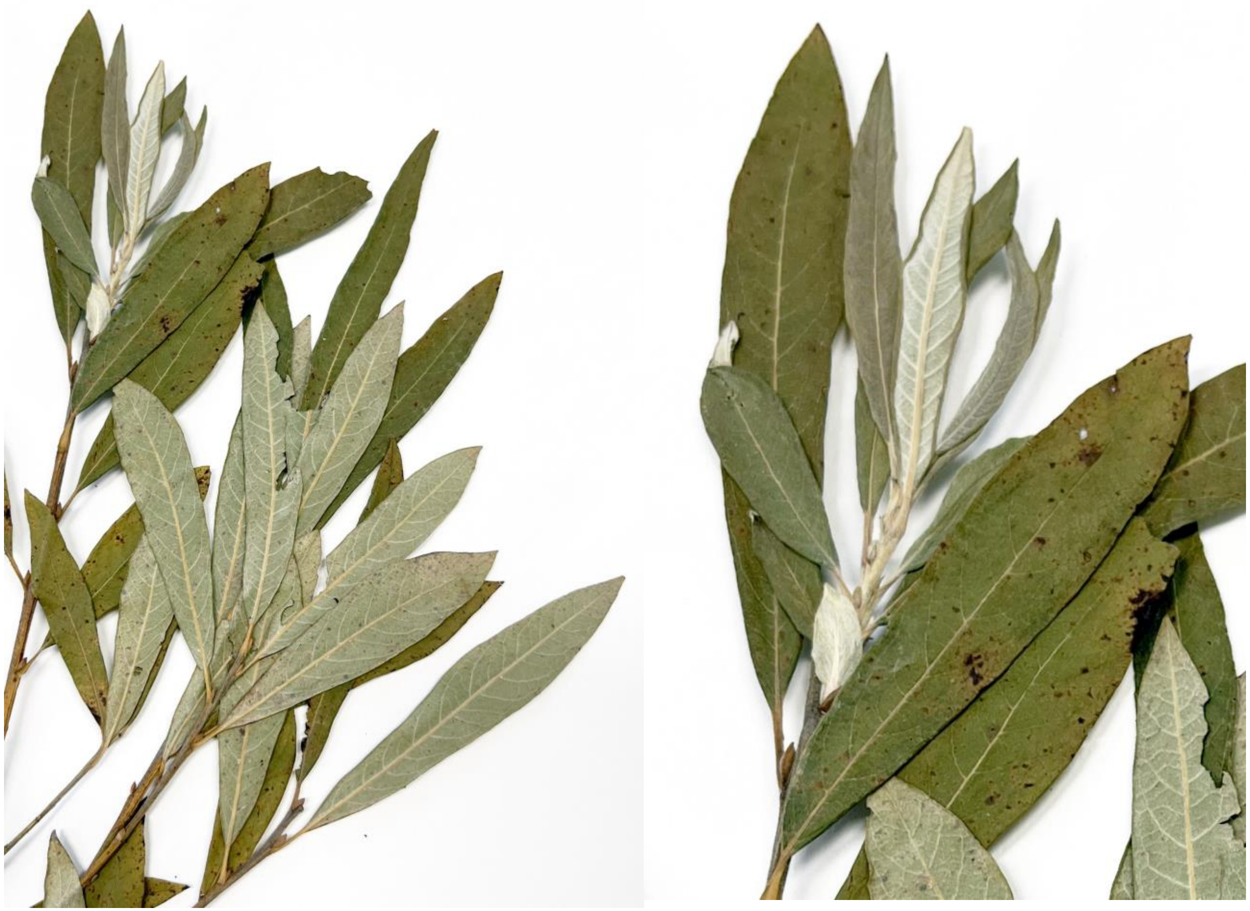

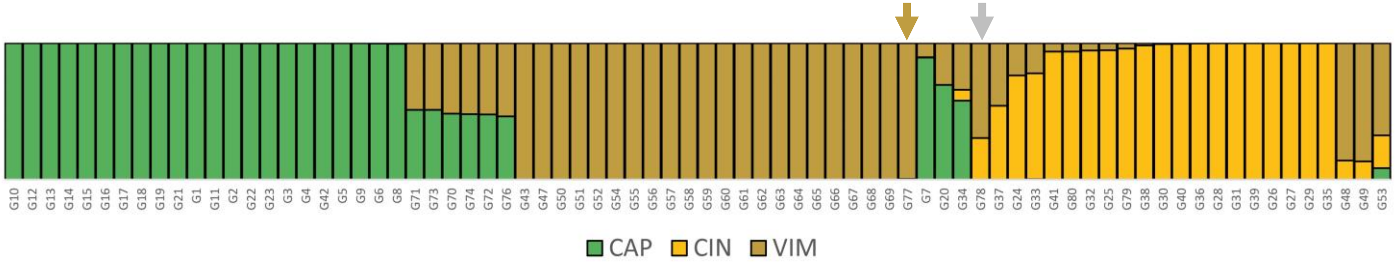

Supplement: Supplementary file 1 [file plants-13-00639-s001.zip › Figures S2.pdf]
