# Supplementary material for: Detection of Hybrids in Willows (Salix, Salicaceae) Using Genome-Wide DArTseq Markers
Source: Plants (Basel). 2024 Feb 26;13(5):639. doi: 10.3390/plants13050639 (PMC10935248; doi:10.3390/plants13050639)
Supplement: Supplementary file 1 [file Figure S1.pdf › Figure S1.pdf]

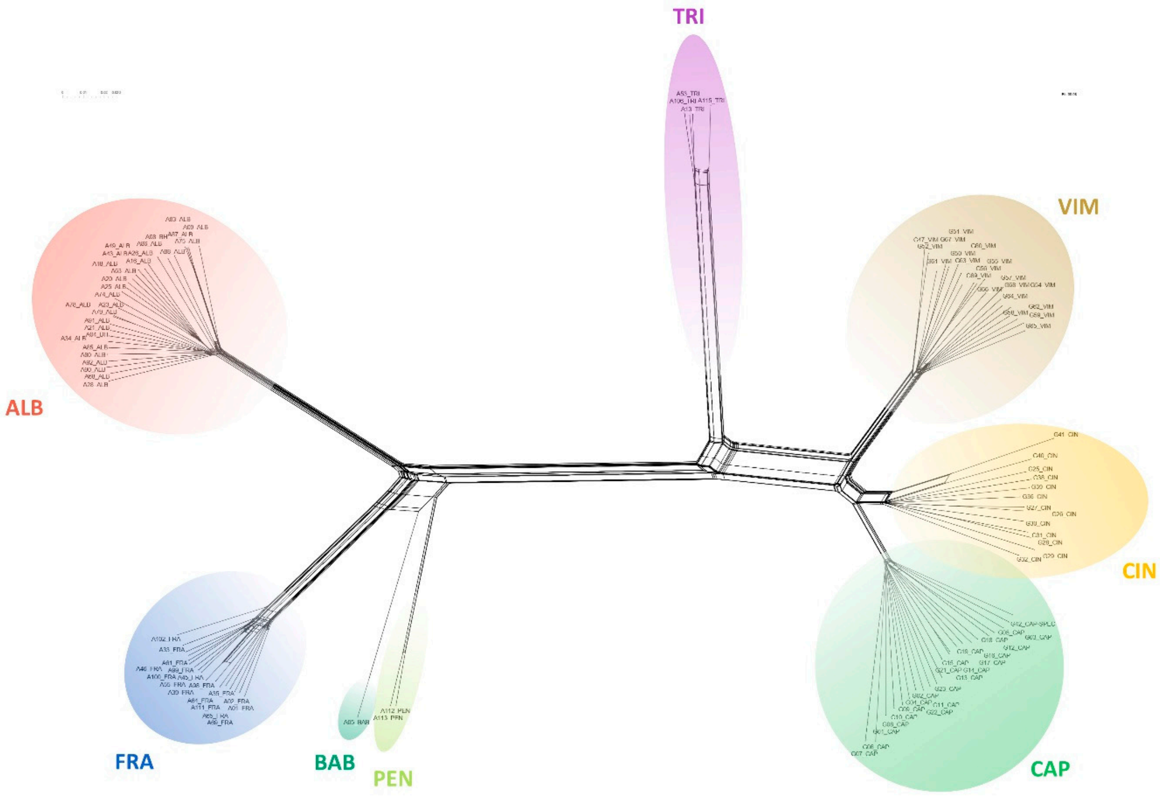

Figure S1. Neighbour-Joining unrooted dendrogram for *Salix* subgen. *Vetrix* and *Salix* subgen. *Salix* based on 185701 binary SNPs generated in SPLITSTREE. Legend to taxa: **CAP** = *S. caprea*; **CIN** = *S. cinerea*; **VIM** = *S. viminalis*; **ALB** = *S. alba*; **FRA** = *S. fragilis*; **TRI** = *S. triandra*; **PEN** = *S. pentandra*; **BAB** = *S. babylonica* 'Babylon'. Taxa of each subgenus cluster together (one group cluster formed by *S. caprea*, *S. cinerea*, *S. viminalis*, second one by *S. alba*, *S. fragilis*, *S. pentandra* and *S. babylonica*), with exception of *S. triandra* that has distant position from both, being closer to taxa of subgenus *Vetrix*.
